# Interaction between karst terrain and bauxites: evidence from Quaternary orebody distribution in Guangxi, SW China

**DOI:** 10.1038/s41598-017-12181-1

**Published:** 2017-09-19

**Authors:** Lin Yang, Qingfei Wang, Qizuan Zhang, Emmanuel John M. Carranza, Huan Liu, Xuefei Liu, Jun Deng

**Affiliations:** 1 State Key Laboratory of Geological Processes and Mineral Resources, China University of Geosciences, Beijing, 100083 China; 2 The Bureau of Geo-exploration Guangxi and Mineral Development, Nanning, 530023 China; 3 State University of Campinas (UniCamp), Campinas, São Paulo Brazil; 4 Geological Sciences, School of Agricultural, Earth and Environmental Sciences, University of KwaZulu-Natal, Durban, South Africa; 5 Economic Geology Research Centre (EGRU), James Cook University, Townsville, Australia

## Abstract

Most bauxite in China is located upon the karst surface, yet the relation between karstification process and bauxite formation is barely known. Here we discuss how the relation affects the karst and bauxite evolution through analyzing distributions of orebody parameters from 9,007 exploration wells (434 orebodies) in western Guangxi, South China block. In high-elevation karst terrain dominated by peaks, orebodies have greater average thickness, lower Al_2_O_3_ and higher Fe_2_O_3_^T^ than those in low-elevation region dominated by depressions. Principal component and multifractal analyses show that the Al_2_O_3_, Fe_2_O_3_^T^ and LOI and the orebody thickness, determined by depression geometry, have more even distributions in high-elevation terrain. This explains that the interaction between the oxidized, alkaline water in karst surface and the ferrous clay minerals that released H^+^ during bauxite secondary weathering was more intensive in high-elevation terrain than in low-elevation one. The interaction with self-organized nature is considered responsible for the even development of karstic depressions and bauxite orebody thicknesses in high-elevation terrain. In comparison, SiO_2_ distribution is more even in low-elevation terrain, where connected depressions near the phreatic zone facilitated SiO_2_ mobilization and even distribution.

## Introduction

Bauxites are traditionally considered to form via intense chemical weathering in hot and humid zones^1^. Bauxite deposits formed on paleokarstic surfaces of carbonates are identified with the karstic category^2–5^, which provides us a unique chance to detect the relation between karstification process and bauxite formation. The karstic bauxites in the western Guangxi, southwestern South China block, can be further divided into two types according to their genesis. The first type is the Permian bauxite deposit, which formed by sedimentation upon the unconformity between the Middle Permian Maokou Formation and the Upper Permian Heshan Formation^6,7^. The second type belongs to the Quaternary bauxite deposit^2^, occurring in the Quaternary karst depressions^8,9^.

The Quaternary bauxites in the western Guangxi have great economic value with verified reserves of more than 0.5 billion tons^7,9^. It is traditionally acknowledged that the Permian bauxites directly transformed into Quaternary bauxites through break-up, secondary weathering and re-sedimentation^7,9–11^. The genesis of the Quaternary bauxites is a unique process that involved not only the formation of bauxite but also the renovation of karst topography^12^. Abundant researches on the Quaternary bauxites have been carried out, mainly focusing on mineralogical and geochemical features^8–10,13–16^, mineralogical change and elemental migration during bauxitization or bauxite transformation^11,14,15^, ore quality and vertical structure^9^. Despite these researches, the distribution and controls of Quaternary bauxites have not yet been studied in detail and especially the relation between karstification process and Quaternary bauxite formation was obviously ignored, although the reaction of bauxites or terra rossa with carbonates has been verified^17–21^.

Here we characterize the orebody distribution of Quaternary bauxites and chemical process during bauxite transformation based on field observations and statistical analyses, including inverse distance weighting (IDW) interpolation, multifractal analysis and principal component analysis, of orebody parameters from 9,007 exploration wells (434 orebodies) in western Guangxi. Accordingly, we propose that the interaction between karstic surface and primary bauxites (Permian bauxites) with self-organized nature is responsible for the even development of Quaternary bauxite orebody in high elevation terrain.

**Figure 1:**
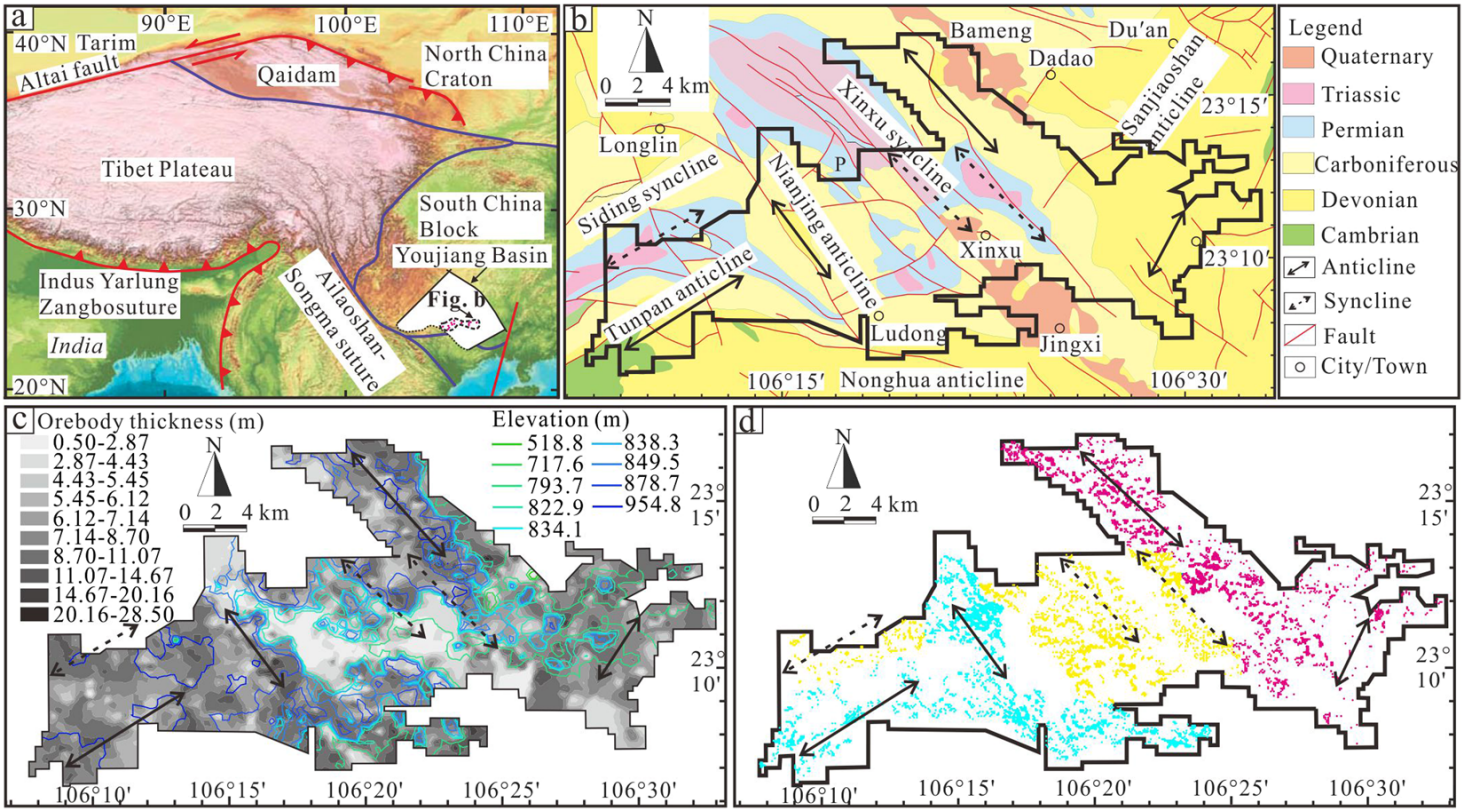
(**a**) Tectonic map showing the locations of the Youjiang basin and study area (modified from Liu *et al*.^11^). (**b**) Simplified geological map showing the structures of study area. (**c**) Contour maps of orebody thickness and elevation. (**d**) Distribution of exploration wells. The bluish, yellow and magenta points represent the exploration wells in western anticline, syncline and eastern anticline areas, respectively. (The contour and drill-hole distribution maps are produced by ArcGIS 10.0 (http://www.esrichina.com.cn/softwareproduct/ArcGIS/)).

**Figure 2:**
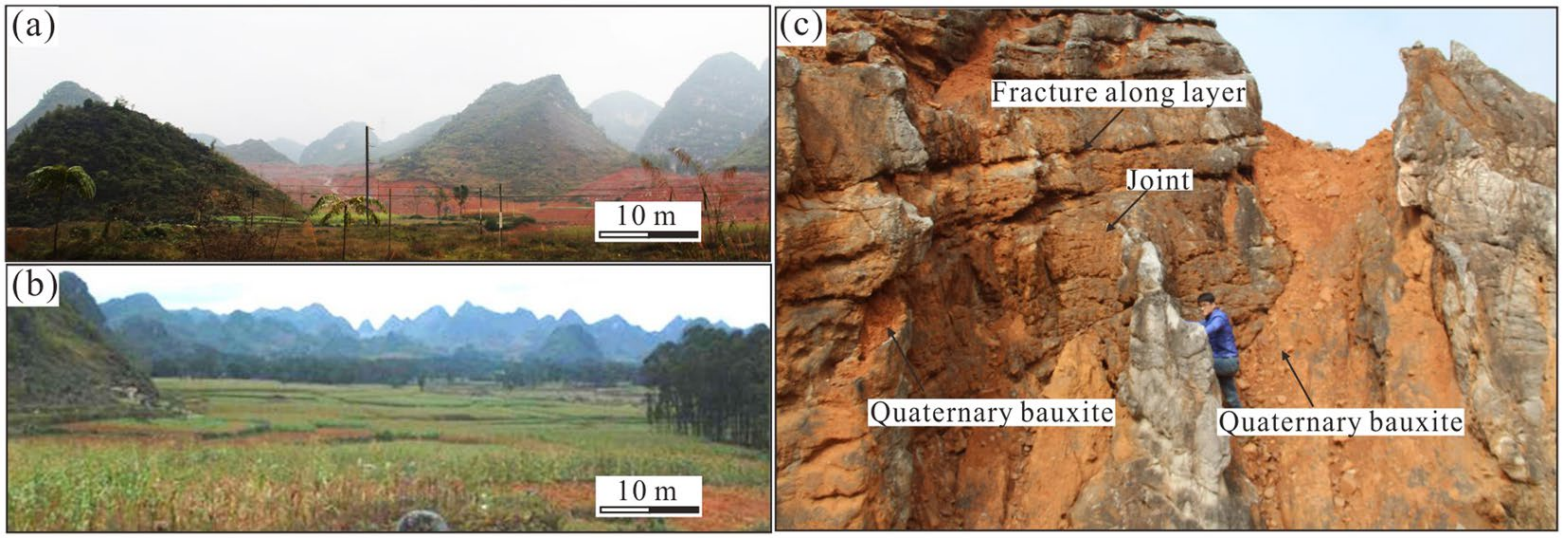
Photographs of karst topography showing (**a**) morphology of peak-dominated terrain, (**b**) morphology of depression-dominated terrain and (**c**) relation between karstification and bauxite.

The western Guangxi paleogeographically belongs to the southwestern quadrant of the Youjiang Basin located along the southwestern margin of the South China block^15^ (Fig. 1a). The exposed stratigraphic sequence, from oldest to youngest, in the western Guangxi mainly consists of Cambrian, Devonian, Carboniferous, Permian, Triassic, Jurassic, Cretaceous, Miocene and Quaternary^9^ (Fig. 1b). The structural framework in the study area is dominated by the NW- and NE-trending folds and faults (Fig. 1b). The anticlines, occurring in the westernmost, eastern and northern parts of the study area, have relative high elevation and the synclines occurring in the westernmost and central parts have both high and low elevation areas (Fig. 1b and c). The karst topography comprises peak- and depression-dominated terrains based on field observations (Fig. 2a and b). The peak-dominated terrain is characterized by the pattern that many peaks and depressions alternatively cluster, and the depressions are relative narrow and close because of small distances between peaks (Fig. 2a). In contrast, the depression-dominated terrain has fewer peaks and is characterized by wide and sometimes connected depressions (Fig. 2b). The residual carbonates in the Quaternary bauxite profile are commonly cut by joints or interstratified cracks, which were filled by Quaternary bauxites, showing an intermediate contact between karst topography and bauxites (Fig. 2c).

## Results

### Orebody distribution

**Figure 3:**
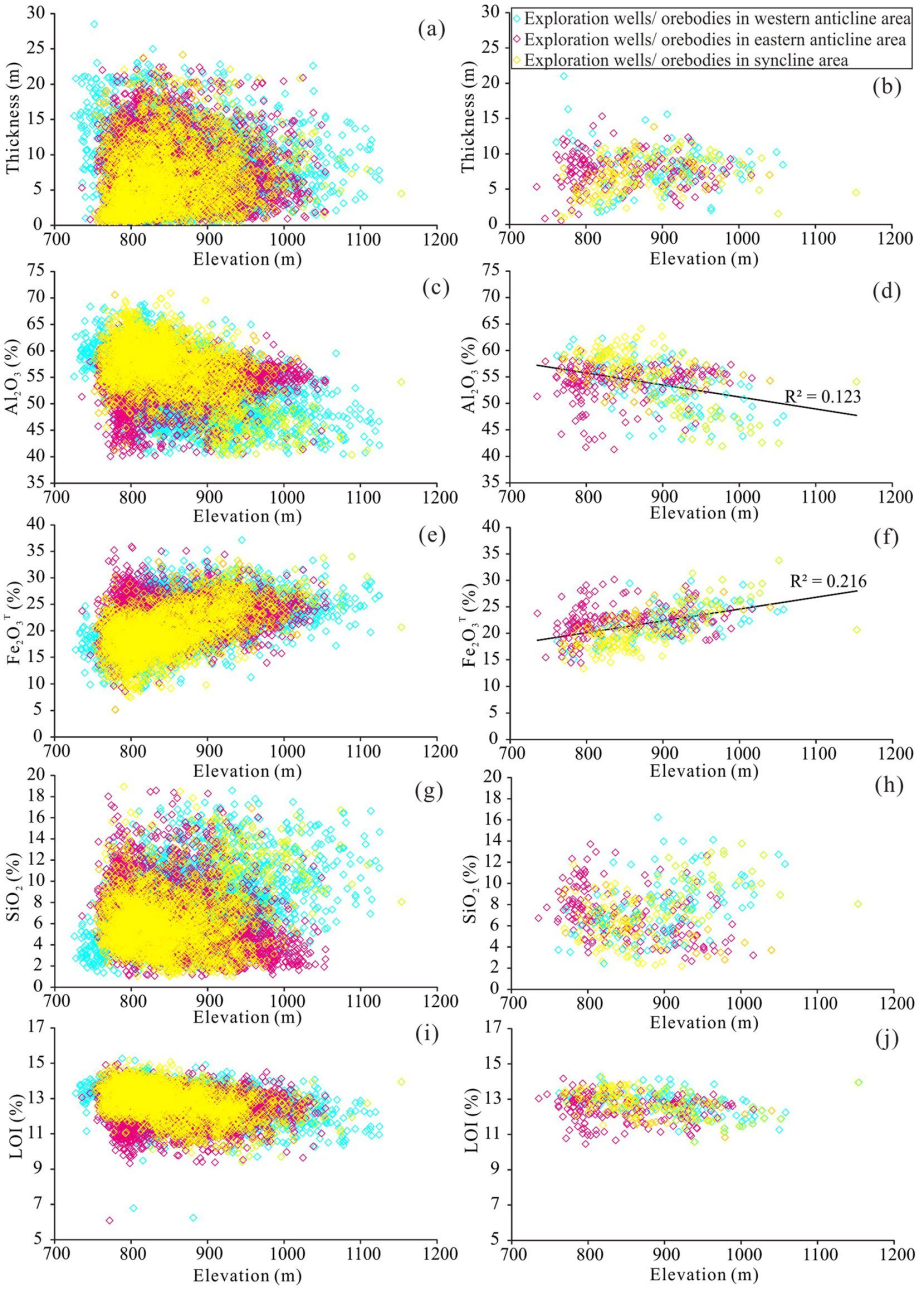
Correlations between thickness and elevation (**a**) and (**b**), Al_2_O_3_ and elevation (**c**) and (**b**), Fe_2_O_3_^T^ and elevation (**e**) and (**f**), SiO_2_ and elevation (**g**) and (**h**), and LOI and elevation (**i**) and (**j**) in 9,007 exploration wells and 434 orebodies, respectively. The bluish, yellow and magenta rhombuses represent the exploration wells or orebodies in western anticline, syncline and eastern anticline areas, respectively.

Based on 9,007 exploration wells from 434 Quaternary bauxite orebodies in the western Guangxi, contour maps of karstic depression elevation and orebody thickness were obtained by IDW interpolation (Fig. 1c). The distribution of exploration wells was shown in Fig. 1d. Quaternary bauxites are generally distributed in karst depressions and are directly in contact with the underlying carbonates, and thus the contour map of underlying elevation of Quaternary bauxites can represent the morphology of ore-bearing karst depressions. By examining the geological and contour maps together, it is recognized that the thicker orebodies generally overlap with the core of anticlines and synclines at high elevation area, whereas the low-elevation synclines commonly contain thinner orebodies (Fig. 1b and c). The distribution of peaks and depressions shows that the high- and low-elevation areas are characterized by peak- and depression-dominated terrains (Fig. 1b). The high-elevation area has greater average thickness (average 7.93 m in high-elevation area and 5.80 m in low-elevation area in 9,007 exploration wells) (Fig. 3a and b), lower Al_2_O_3_ (Fig. 3c and d) and higher Fe_2_O_3_^T^ (Fig. 3e and f) than those in the low-elevation one based on the statistics of orebody parameters and elevation in the exploration wells and orebodies. The correlations between elevation and orebody thickness, elevation and Al_2_O_3_, elevation and Fe_2_O_3_^T^, elevation and SiO_2_ and elevation and LOI in 9,007 exploration wells (Fig. 3a,c,e,g and i) and those between elevation and ore thickness, elevation and SiO_2_ and elevation and LOI in 434 orebodies (Fig. 3b,h and j) are weak, while those between elevation and Al_2_O_3_ and between elevation and Fe_2_O_3_^T^ in 434 orebodies are strong (Fig. 3d and f).

**Figure 4:**
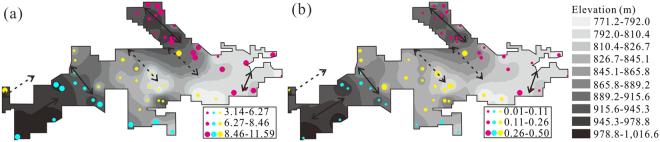
Spatial distributions of (**a**) orebody thickness and (**b**) Δα of orebody thickness of 55 selected orebodies, with overlay of elevation contours. (The contour maps are produced by ArcGIS 10.0 (http://www.esrichina.com.cn/softwareproduct/ArcGIS/)). The bluish, yellow and magenta circles represent the orebodies in western anticline, syncline and eastern anticline areas, respectively.

The spatial distributions of orebody thickness, major chemical components and elevation of 55 large orebodies are shown in Figs 4 and S1 (“S” means Supplementary materials). Orebodies thicker than 8.46 m are distributed in the western and eastern anticline areas (Fig. 4a). These parts of the study area commonly have higher elevations compared to the other parts with low elevations where orebodies tend to be thinner (Fig. 4a). Values of Al_2_O_3_ > 55.81% appear in the syncline area where elevation is low; other parts of the area have lower Al_2_O_3_ (Fig. S1a). The distribution of Fe_2_O_3_^T^ is opposite to that of Al_2_O_3_, meaning that parts of the area with high Al_2_O_3_ have a low Fe_2_O_3_^T^ and vice versa (Fig. S1b). Values of SiO_2_ > 7.77% mainly occur in the western and eastern anticline areas (Fig. S1c). The distribution of LOI values shows no obvious regular pattern with elevation (Fig. S1d).

The spatial distributions of elevation and orebody parameters in the single orebodies XX21 and XX20 are shown in Fig. S2. The orebody XX21 has a great average thickness of 11.59 m and occurs in the high elevation of 863.9 m while the XX20 with the thickness of 3.86 m appears in the altitude of 787.3 m (Table S1). The spatial distributions of orebody parameters of the two single orebodies are consistent with that of 55 orebodies overall (Figs 4, S1 and S2), but some differences still exist. For example, great values of Al_2_O_3_ distribute in the high altitude area in the orebody XX21 (Fig. S2a) and those of Fe_2_O_3_^T^ occur in the low altitude area in the orebody XX20 (Fig. S2f). The differences may depend on the observation scale determining the variant elevation, i.e., the high-elevation depressions in a small scale may correspond to low-elevation ones in the big scale.

### Multifractal parameter distributions

The multifractal parameters of selected 55 orebodies can be seen in Table S1. The styles of multifractal spectra of orebody thickness, Al_2_O_3_, Fe_2_O_3_^T^, SiO_2_ and LOI for thick orebody XX21 and thin orebody XX20 are illustrated in Fig. S3. The values of Δα for thickness and major chemical components of 55 selected orebodies are displayed in the horizontal projection plane, over which the elevation contour map is shown (Figs 4 and S4). Large values of Δα for orebody thickness (>0.26) are mainly distributed in the central syncline with low-elevation, whereas small values of Δα for orebody thickness occur in the western and eastern anticline areas with high-elevation (Fig. 4b). The values of Δα for Al_2_O_3_ are dominated by small values and the largest value is less than 66.4 × 10^−4^. The larger values of Δα (>17.8 × 10^−4^) for Al_2_O_3_ are distributed in the western and eastern anticline areas with relative low elevation, while the smaller values of Δα (<4.0 × 10^−4^) for Al_2_O_3_ are distributed in the western and eastern anticline areas with high-elevation (Fig. S4a). The distribution of Δα values for Fe_2_O_3_^T^ is similar with that of Δα values for Al_2_O_3_ (Fig. S4b). However, the distribution of Δα values for SiO_2_ is almost opposite to that for Al_2_O_3_ and Fe_2_O_3_^T^ (Fig. S4c). The values of Δα for LOI occur in syncline and eastern anticline with low and high elevation, respectively, and barely show correlation with elevation (Fig. S4d).

**Figure 5:**
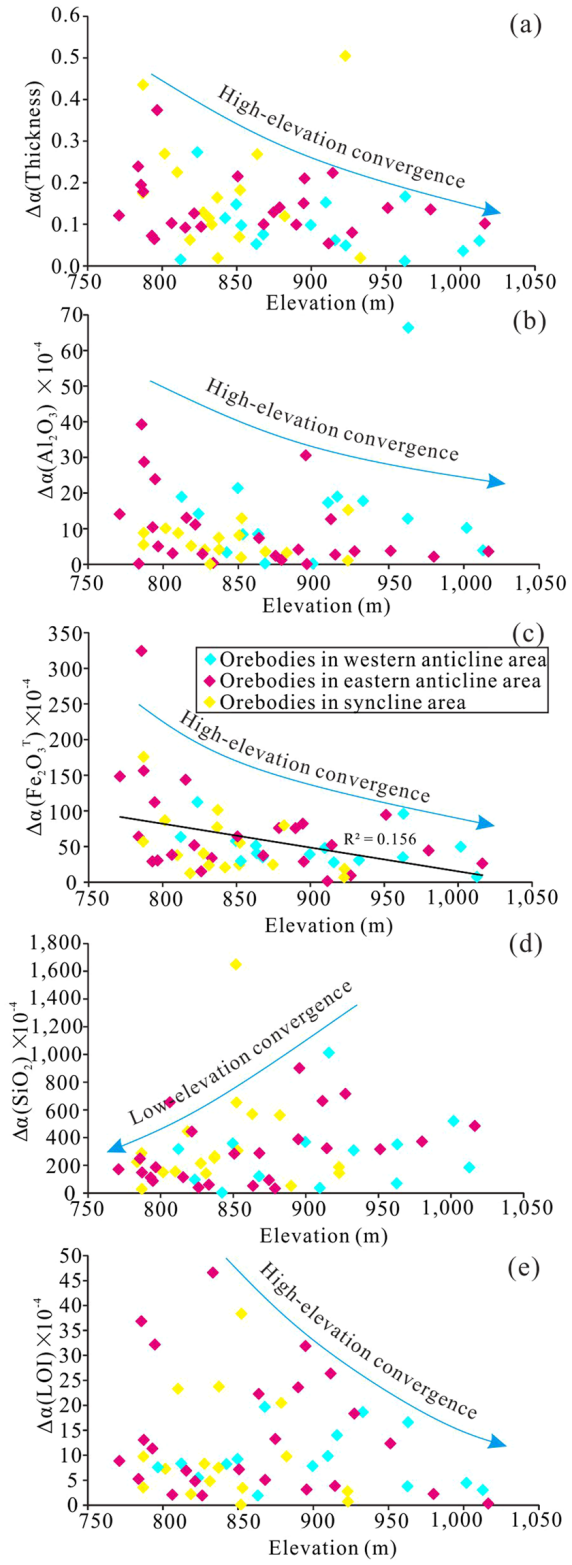
Correlations of elevation with values of Δα in 55 selected orebodies in the western Guangxi, China for: (**a**) thickness, (**b**) Al_2_O_3_, (**c**) Fe_2_O_3_^T^, (**d**) SiO_2_ and (**e**) LOI. The values of Δα for orebody thickness, Al_2_O_3_, Fe_2_O_3_^T^ and LOI show a trend of convergence towards high elevation, whereas those of Δα for SiO_2_ show a trend of convergence towards low elevation. The bluish, yellow and magenta rhombuses represent the orebodies in western anticline, syncline and eastern anticline areas, respectively.

The values of Δα for orebody thickness, Al_2_O_3_, SiO_2_ and LOI show little correlations with elevations (Fig. 5a,b,d and e), whereas elevations and values of Δα for Fe_2_O_3_^T^ display a clear negative correlation (Fig. 5c). Despite the weak correlations between elevations and Δα of different parameters, the values of Δα for orebody thickness, Al_2_O_3_, Fe_2_O_3_^T^ and LOI tend to decrease as elevation increases (Fig. 5a,b,c and e), whereas those for SiO_2_ tend to increase with increasing elevation (Fig. 5d).

### Associations of elevation and orebody parameters

Three significant PCs of elevation, orebody thickness, ore-bearing rate, Al_2_O_3_, Fe_2_O_3_^T^ and SiO_2_, LOI, A/S and A/F from 9,007 wells were obtained through PCA (Table S2). The PC1 (accounting for 42.89% of the total variance with an eigenvalue of 3.86) represents antipathetic association of Fe_2_O_3_^T^ and thickness (with negative loadings of −0.93 and −0.64, respectively) with A/F, Al_2_O_3_ and LOI (with positive loadings of 0.91, 0.81 and 0.67, respectively). The PC2 (with an eigenvalue of 1.71 accounting for 19.02% of the total variance) represents antipathetic association between SiO_2_ (with negative loading of −0.88) and A/S (with positive loading of 0.88). The PC3, accounting for 11.17% of the total variance with an eigenvalue of 0.96, represents elevation with positive loading of 0.95.

The PCA of elevation and orebody parameters for high- and low-elevation wells and single orebody XX21 and XX20 yielded three PCs (Table S2). The PC1 for high-elevation wells (accounting for 41.07% of the total variance with an eigenvalue of 3.70) represents antipathetic association of Fe_2_O_3_^T^ (with negative loading of −0.96) with A/F and Al_2_O_3_ (with positive loadings of 0.94 and 0.75, respectively). The PC2 (with an eigenvalue of 1.60 accounting for 17.74% of the total variance) represents antipathetic association of SiO_2_ (with negative loading of −0.94) with A/S and Al_2_O_3_ (with positive loadings of 0.92 and 0.62, respectively). The PC3 (accounting for 12.47% of the total variance with an eigenvalue of 1.12) represents an association of elevation and ore-bearing rate with positive loadings of 0.73 and 0.71, respectively (Table S2).

The low-elevation wells and single orebody XX21 and XX20 have the same PC1 (antipathetic association of Fe_2_O_3_^T^ with A/F, Al_2_O_3_ and LOI) and PC2 (antipathetic association of SiO_2_ with A/S) despite the different variances, eigenvalues and loadings, which can be seen in Table S2. The PC3 for low-elevation wells (accounting for 11.72% of the total variance with an eigenvalue of 1.05) represents an association of thickness and ore-bearing rate with positive loadings of 0.82 and 0.69, respectively (Table S2). The PC3 (with an eigenvalue of 1.73 accounting for 19.21% of the total variance) for single orebody XX21 represents antipathetic association of elevation with thickness with negative and positive loadings of −0.81 and 0.78, respectively. The PC3 (with an eigenvalue of 1.34 accounting for 15.38% of the total variance) for single orebody XX21 represents an association of thickness and elevation with positive loadings of 0.85 and 0.66, respectively (Table S2).

## Discussion

Although results of PCA for different observation scales and elevations have certain differences in parameter associations between elevation, thickness, chemical components, and ore-bearing rate, they all reflect two independent geochemical processes for bauxite transformation in the western Guangxi, namely (1) enrichment of Al_2_O_3_ and LOI and depletion of Fe_2_O_3_^T^ reflected by the positive and negative loadings, respectively, and (2) depletion of SiO_2_ as supported by the negative loading. Changes in the chemical components support the views of Mameli *et al*.^5^ and Wang *et al*.^9^. Combined with mineral transformation in Quaternary bauxites described in literature, the enrichment of Al_2_O_3_ and LOI was related to the disaggregation of chamosite/chlorite followed by precipitation of Al-oxyhydroxides, such as diaspore/boehmite^8,22^. The chamosite/chlorite formed under a reducing and alkaline environment in Permian, as it is unstable and readily soluble in acidic and oxidizing weathering conditions^8,10,23^. The disintegration of chamosite/chlorite releases aluminum and ferrous ions, silica and H_2_O^10,24^. A possible explanation for Fe_2_O_3_^T^ depletion, together with Al_2_O_3_ enrichment, is that Al ions substituted Fe ions in goethite, a process which has been proved by Boulangé *et al*.^25^ and Laskou *et al*.^26^, and the leach of Fe out of the system. The SiO_2_ depletion implies that the process involved Si release from chamosite/chlorite disaggregation and then Si loss due to few newly-formed Si-bearing minerals, such as kaolinite^8^.

**Figure 6:**
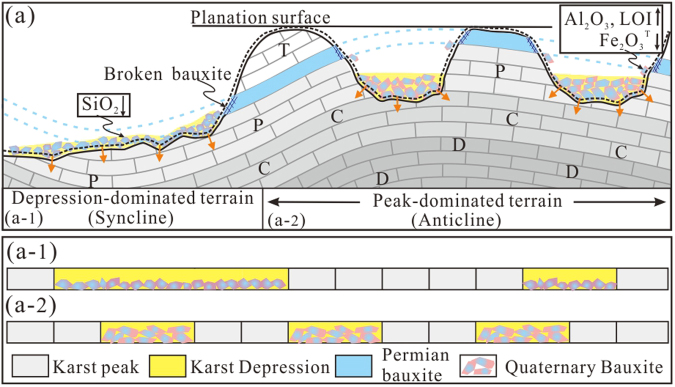
(**a**) Schematic diagram showing bauxite transformation and interaction of bauxites and carbonates. The bauxite transformation in high-level peak-dominated terrain (commonly anticlines) was dominated by depletion of Fe_2_O_3_^T^ (black down arrow) and enrichment of Al_2_O_3_ and LOI (black up arrow), whereas that in low-level terrain (commonly synclines) was dominated by SiO_2_ depletion (black down arrow). Orange arrows mean the interaction of bauxites and carbonates. Black dotted line represents the boundary line between bauxites and carbonates in certain time before. Blue dotted line represents the reacted primary bauxites. Schematic diagram showing the distribution of karst depressions and bauxites in the low- (a-1) and high-level terrain (a-2). The distribution pattern is inferred by the results of multifractal analysis of orebody thickness (See text for more explanation).

The values of Δα for orebody thickness, Al_2_O_3_, Fe_2_O_3_^T^ and LOI show a trend of convergence towards high elevation (Fig. 5a–c and e), meaning that they have more even distribution in high-elevation terrain while Fe_2_O_3_^T^, Al_2_O_3_ and LOI were involved in a single geochemical process. This signature explains that the bauxite transformation in high-elevation terrain was dominated by depletion of Fe_2_O_3_^T^ and enrichment of Al_2_O_3_ and LOI (Fig. 6a). Despite Fe_2_O_3_^T^ depletion during bauxite transformation, the Fe_2_O_3_^T^ content is still high due to the oxidized conditions at high karst depressions^9^. The evenness of these parameters may result from the even-distribution depressions in the high elevation terrain as evidenced by the elevation contour and field observation (Figs 1c and 2a). Although traditional control factors, such as acid rain and underground water system, may play a role for karst topography^27,28^, it is hard to explain the genesis of even-distribution karst depressions in high-level terrain based on these factors. The reaction of karst with overlying bauxites with self-organization mechanism has been verified^17,19,21^. The self-organization mechanism means that the various components of the system of interest spontaneously reacted in a purposeful (non-random) manner, such as the resultant regular distribution of element and geomorphology^29^. The interaction between the oxidized, alkaline water in karst terrain and the ferrous clay minerals that released H^+^ during bauxite secondary weathering^7,8,10,15^ (Fig. 6a) was more intensive in high-elevation terrain than low-elevation one. The intensive interaction with self-organized nature was considered responsible for the even development of karstic depression and bauxite orebody^17,19^. The interaction constitutes a positive feedback due to acid release during bauxite secondary transformation and acid consumption during karstification, which drove the coevolution of karst and bauxites.

The values of Δα for SiO_2_ show a contradictory trend of convergence towards low elevation (Fig. 5d), implying that the distribution of SiO_2_ was more even in low karst depressions. The fact that SiO_2_ was involved in the other dependent chemical process, together with its even distribution, suggests that bauxite transformation in the low karst depressions was dominated by SiO_2_ depletion (Fig. 6a). These signatures imply that the more mobile elements have greater evenness of spatial distribution. The high-level karst terrain near vadose zone often has a high groundwater table and oxidizing conditions while the low-level karst terrain close to phreatic zone has a relatively low groundwater table and reducing conditions^9,12^. The more phreatic condition in lower elevation depression facilitated SiO_2_ mobilization^8,10^ and even distribution.

The peak-dominated depressions in the high-elevation anticline and syncline area contain thick orebodies, whereas the low-elevation synclines contain thin orebodies (Fig. 1c). Meanwhile, the anticline and syncline areas have indistinguishable orebody parameters and Δα values in the exploration wells and bauxite orebodies (Figs 3, 4, S1 and S4). These signatures suggest that the anticlines and synclines had no (or had very little) control on the distribution of Quaternary bauxites. The uniform correlation between the elevation and ore geochemical content in the various structures means that elevation, compared to structures, took the predominant control on the Quaternary ore grade. Continuous weighting of evidence layers for prospectivity analysis^30,31^ can thus be abstracted into two aspects. One aspect is elevation and the other aspect is the structural location where the primary orebody has been exposed and experienced subsequent transformation; with the former aspect having stronger relative importance than the latter aspect.

## Methods

### Data acquisition

Exploration of the bauxite deposit was carried out by the Bureau of Geo-exploration and Mineral Development of Guangxi. During exploration, samples with 1–m length were collected equidistantly and continuously along the bauxite profile. The elevation and orebody parameters (orebody thickness, ore-bearing rate (proportion of industrial recoverable part in whole orebody), Al_2_O_3_, Fe_2_O_3_^T^, SiO_2_, LOI, A/S (ratio of Al_2_O_3_/SiO_2_) and A/F (ratio of Al_2_O_3_/Fe_2_O_3_^T^)) were tested in each exploration well. The major chemical components of each sample were determined by EDTA titrimetric method, orthophenanthroline photometric method, gravimetric-molybdenum blue photometric method and gravimetric method, respectively^9^. Elevation and orebody parameters of 434 orebodies (9,007 wells) of Quaternary bauxites overlapping the limestones in the western Guangxi were collected for this work.

### Statistical methods

Based on 9,007 exploration wells, the elevation surface of depressions was obtained via IDW interpolation (by ArcGIS 10.0) with the following parameters: search radius of 397 m, weighting power of 2, maximum of 15 samples per window, and interpolation interval of 10 m. Similarly, contour maps of orebody thicknesses (from 9,007 exploration wells) and those of thickness Al_2_O_3_, Fe_2_O_3_^T^, SiO_2_ and LOI (from selected 55 orebodies containing >20 exploration wells in each orebody) were obtained to show their spatial distributions. The multifractal parameters of orebody thickness and major chemical components in the selected 55 orebodies were calculated through the multifractal model with box-gliding method (BGM) as described by Cheng^32^.

In the BGM, a box of a certain side dimension (*ε*) was glided over the data of a variable in each exploration well to estimate the spatial distributions of thickness and major chemical components. Such an approach has been widely applied to analyze skewed geological data by means of multifractal spectrum^33–36^, composed by the α(q) (called singularity exponent) and the corresponding f(α) (called fractal dimension), with inverse bell shape^37^. The calculation procedure for this model can be found in the Supplementary Materials of Yang *et al*.^38^. The multifractal spectrum was calculated with q (called moment) ranging from −1 to 1. The Δα (the width of a multifractal spectrum) and the Δf(α) (the height difference between right and left terminals of a multifractal spectrum) were used to characterize the spectrum. A decrease in Δα indicates a tendency to a more even distribution^35^.

### Principal component analysis

To unravel the correlations among the elevation and orebody parameters of Quaternary bauxites, PCA for 9,007 wells, high- and low-elevation wells (divided by mean value of elevations), thick single orebody XX21 and thin single orebody XX20 were performed. Only principal components (PCs) with eigenvalues greater than 1 were extracted based on the Kaiser criterion^39^. The extracted PCs were subjected to orthogonal rotation by the varimax method to maximize variability (i.e., to strongly differentiate) among all input variables and thus facilitate interpretation of the factor loadings^39^. The PCA was implemented using software SPSS v.20.

## Electronic supplementary material

Supplementary Information
