# Supplementary material for: Interaction between karst terrain and bauxites: evidence from Quaternary orebody distribution in Guangxi, SW China
Source: Sci Rep. 2017 Sep 19;7:11842. doi: 10.1038/s41598-017-12181-1 (PMC5605576; doi:10.1038/s41598-017-12181-1)
Supplement: Supplementary file 1 — Supplementary Information [file 41598_2017_12181_MOESM1_ESM.doc]

**Interaction between karst terrain and bauxites: evidence from Quaternary orebody distribution in Guangxi, SW China**

**Lin Yang^1^, Qingfei Wang^1^,*, Qizuan Zhang^2^, Emmanuel John M.** **Carranza^3,4,5^, Huan Liu^1^, Xuefei Liu^1^, Jun Deng^1^,***

^1^State Key Laboratory of Geological Processes and Mineral Resources, China University of Geosciences, Beijing 100083, China

^2^The Bureau of Geo-exploration Guangxi and Mineral Development, Nanning 530023, China

^3^State University of Campinas (UniCamp), Campinas, São Paulo, Brazil

^4^Geological Sciences, School of Agricultural, Earth and Environmental Sciences, University of KwaZulu-Natal, South Africa

^5^Economic Geology Research Centre (EGRU), James Cook University, Townsville, Australia

* *To whom correspondence should be addressed. E-mail:* [*wqf@cugb.edu.cn*](mailto:wqf@cugb.edu.cn)*, djun@cugb.edu.cn*


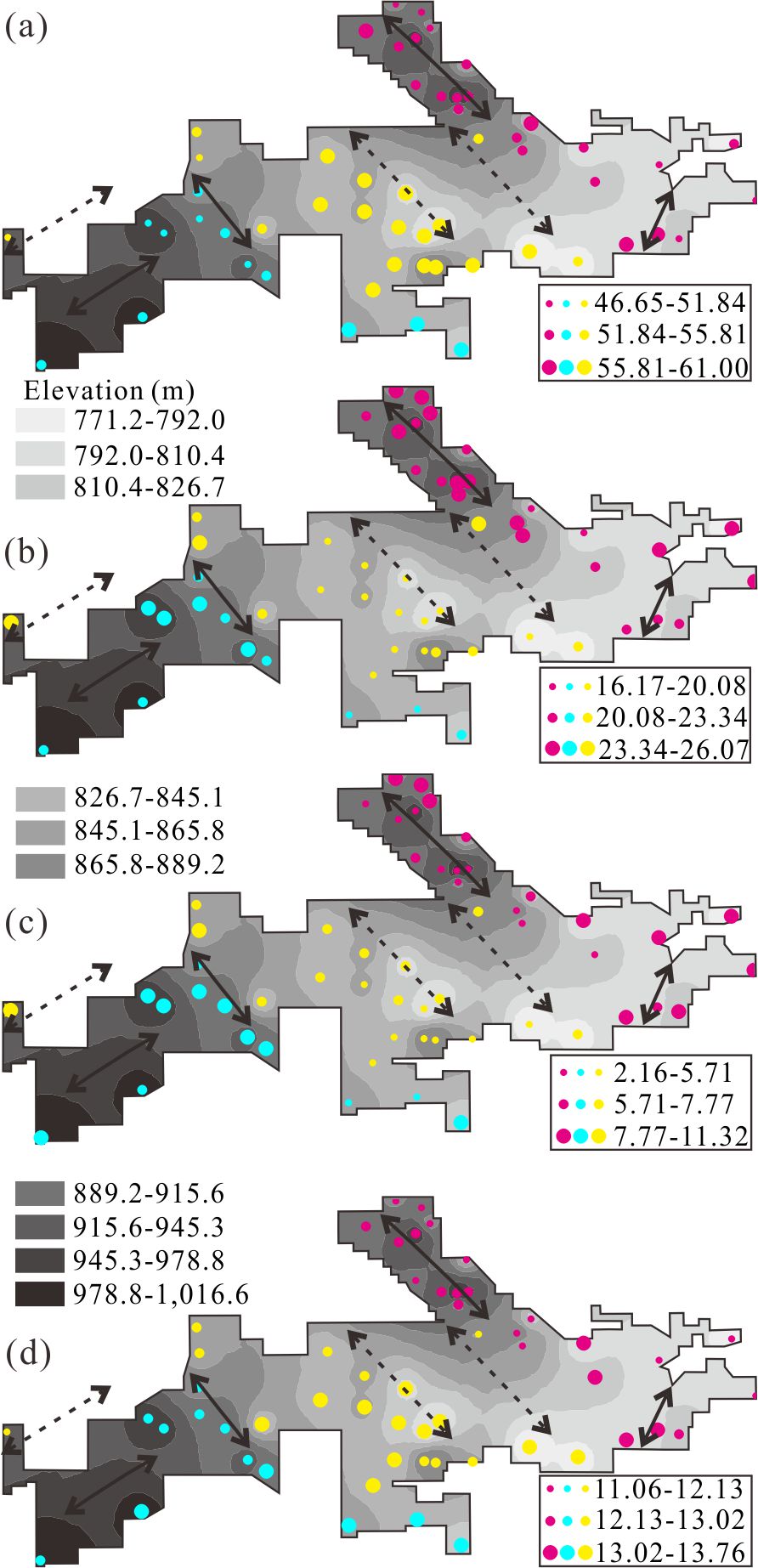


Fig. S1. Spatial distributions of (a) Al_2_O_3_, (b) Fe_2_O_3_^T^, (c) SiO_2_ and (d) LOI of 55 selected orebodies, with overlay of elevation contours (The contour maps are produced by ArcGIS 10.0 (http://www.esrichina.com.cn/softwareproduct/ArcGIS/)). The bluish, yellow and magenta points represent the exploration wells in western anticline, syncline and eastern anticline areas, respectively.


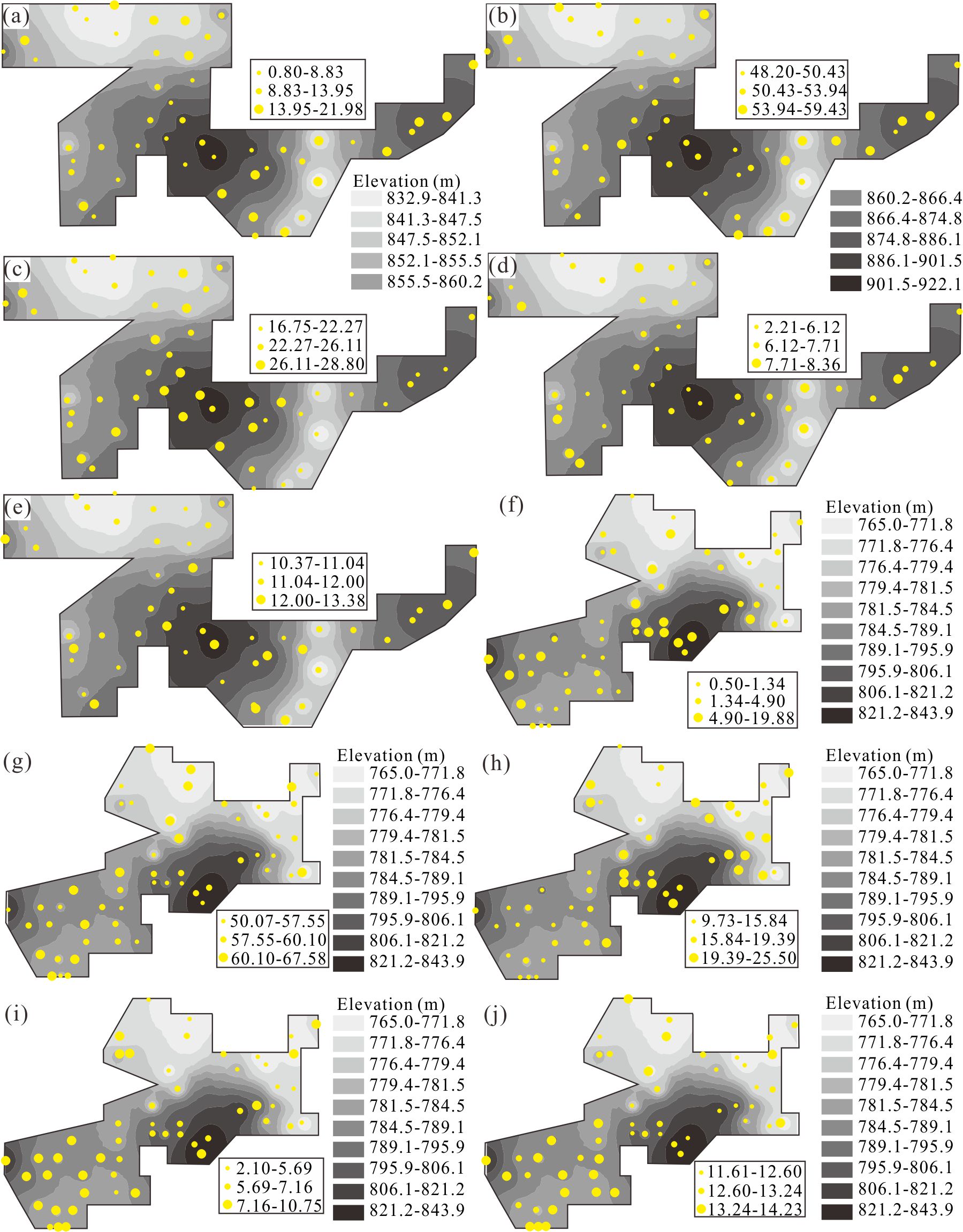


Fig. S2. Spatial distributions of (a) thickness, (b) Al_2_O_3_, (c) Fe_2_O_3_^T^, (d) SiO_2_ and (e) LOI of thick single orebody XX21, with overlay of elevation contours. Spatial distributions of (f) thickness, (g) Al_2_O_3_, (h) Fe_2_O_3_^T^, (i) SiO_2_ and (j) LOI of thin single orebody XX20, with overlay of elevation contours (The contour maps are produced by ArcGIS 10.0 (http://www.esrichina.com.cn/softwareproduct/ArcGIS/)).


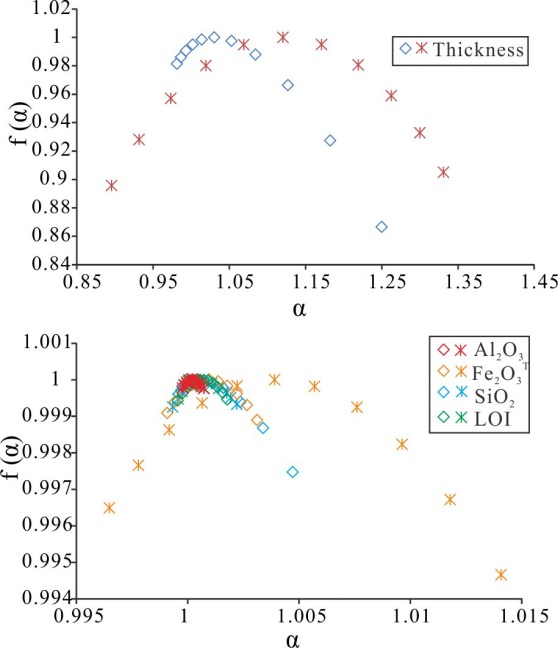


Fig. S3. Overlaps of multifractal spectra of the distributions of thickness and major chemical components in the single XX21 and XX20 orebodies, western Guangxi, southwestern South China block.


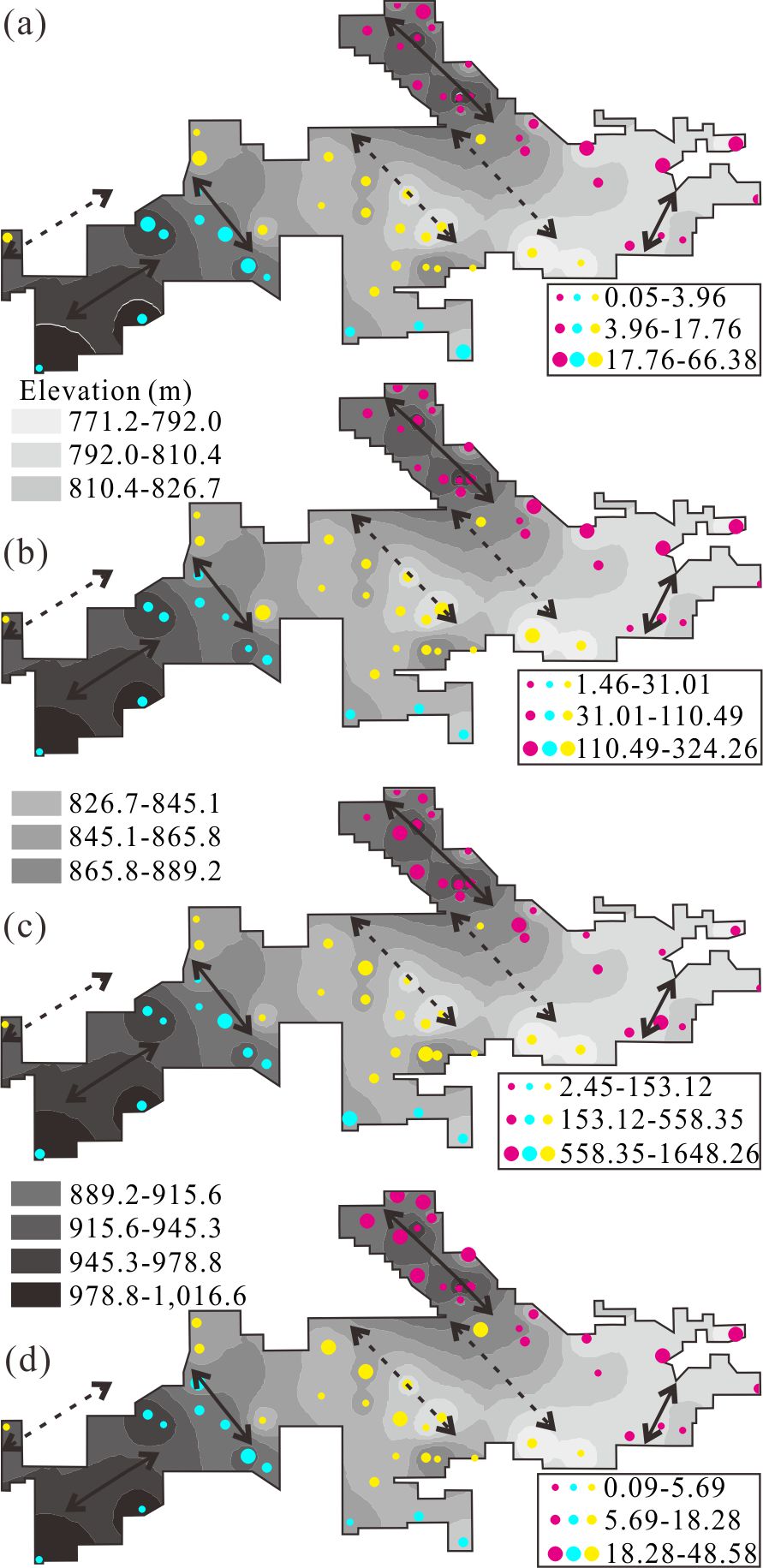


Fig. S4. Spatial distribution of Δα in 55 selected orebodies in the western Guangxi, China for: (a) Al_2_O_3_, (b) Fe_2_O_3_^T^, (c) SiO_2_ and (d) LOI, with overlay of elevation contours (The contour maps are produced by ArcGIS 10.0 (<http://www.esrichina.com.cn/softwareproduct/ArcGIS/)>). The bluish, yellow and magenta points represent the exploration wells in western anticline, syncline and eastern anticline areas, respectively.

Table S1 Means and ranges of elevation, orebody thickness and major chemical components, and multifractal parameters of orebody thickness and major chemical components in the selected 55 orebodies in the western Guangxi, China

|  |  | Thickness | | | Al_2_O_3_ | | | Fe_2_O_3_^T^ | | | SiO_2_ | | | LOI | | |
| --- | --- | --- | --- | --- | --- | --- | --- | --- | --- | --- | --- | --- | --- | --- | --- | --- |
| Orebody | Altitude | Min~Max  (Mean,Var) | Δα ×10^-4^ | Δf (α) ×10^-4^ | Min~Max (Mean,Var) | Δα ×10^-4^ | Δf (α) ×10^-4^ | Min~Max  (Mean,Var) | Δα ×10^-4^ | Δf (α) ×10^-4^ | Min~Max  (Mean,Var) | Δα ×10^-4^ | Δf (α) ×10^-4^ | Min~Max  (Mean,Var) | Δα ×10^-4^ | Δf (α) ×10^-4^ |
| SH119 | 1021.1 | 1.85~13.55 | 599.41 | -18.61 | (47.35, 58.42) | 3.90 | 0.34 | (17.2, 26.31) | 7.55 | 0.37 | (4.19,14.59) | 184.65 | -42.06 | (11.55,13.33) | 3.02 | 0.09 |
|  |  | (7.71,10.95) |  |  | 53.19~9.07 |  |  | 22.33~9.11 |  |  | 7.95~10.40 |  |  | 12.26~1.78 |  |  |
| SH142 | 937.0 | 3.9~19.80 | 490.03 | -82.28 | (40.59, 51.96) | 15.26 | -0.38 | (21.48, 28.46) | 18.47 | 0.04 | (7.49,14.37) | 143.61 | -0.94 | (11.08,12.78) | 0.73 | -0.02 |
|  |  | (11.29~15.9) |  |  | 47.31~11.37 |  |  | 23.44~7.98 |  |  | 11.06~6.88 |  |  | 12.02~1.70 |  |  |
| SH167 | 975.6 | 1.2~22.1 | 1667.0 | 3.78 | (40.17, 63.46) | 66.38 | 2.58 | (13.8, 34.03) | 95.87 | -7.93 | (4.53,14.69) | 350.86 | -51.67 | (11.06,14.12) | 16.61 | 0.10 |
|  |  | (9.29~20.9) |  |  | 48.64~13.29 |  |  | 23.88~21.23 |  |  | 10.76~10.16 |  |  | 12.55~3.06 |  |  |
| SH170 | 974.7 | 2.1~14.7 | 111.88 | -5.55 | (41.76, 56.04) | 12.80 | 0.51 | (17.31, 31.6) | 34.69 | -2.90 | (6.02,15.91) | 68.77 | -0.65 | (11.32,13.94) | 3.77 | 0.04 |
|  |  | (10.47~12.6) |  |  | 47.00~15.28 |  |  | 24.42~14.29 |  |  | 10.35~9.89 |  |  | 12.75~2.62 |  |  |
| SH210 | 941.8 | 1.0~19.2 | 188.69 | 149.06 | (42.07, 55.96) | 17.77 | 0.52 | (21.30, 31.96) | 30.92 | 3.11 | (3.05,13.72) | 307.86 | -62.33 | (11.28,13.98) | 18.61 | 0.02 |
|  |  | (7.48~18.2) |  |  | 47.74~13.89 |  |  | 26.07~10.66 |  |  | 8.68~10.67 |  |  | 12.63~2.70 |  |  |
| SH211 | 912.2 | 2.3~22.8 | 981.05 | -294.96 | (44.34, 57.58) | 0.14 | -0.04 | (17.30, 37.15) | 39.11 | 2.59 | (3.99, 8.45) | 368.68 | 4.19 | (12.43,14.76) | 7.81 | 0.07 |
|  |  | (10.71~20.5) |  |  | 52.62~13.24 |  |  | 22.73~19.85 |  |  | 5.75~4.46 |  |  | 13.35~2.33 |  |  |
| SH248 | 1009.4 | 3.4~11.3 | 357.86 | -42.42 | (49.03, 57.56) | 10.20 | -0.22 | (16.77, 23.59) | 49.49 | -5.27 | (3.99, 12.34) | 518.84 | 3.64 | (12.06,14.01) | 4.46 | 0.10 |
|  |  | (7.14~7.9) |  |  | 53.64~8.53 |  |  | 21.40~6.82 |  |  | 7.69~8.35 |  |  | 13.03~1.95 |  |  |
| LB415 | 875.3 | 0.6~12.96 | 752.79 | 47.99 | (45.1, 58.09) | 0.22 | -0.02 | (18.00, 25.55) | 34.89 | 0.50 | (2.35, 13.64) | 121.41 | -3.87 | (10.17,13.73) | 19.67 | -1.05 |
|  |  | (5.66,12.85) |  |  | 53.86~12.99 |  |  | 21.78~7.55 |  |  | 6.89~11.29 |  |  | 12.42~3.56 |  |  |
| LB452 | 924.3 | 2.06~14.9 | 612.81 | -21.43 | (45.77, 59.80) | 18.98 | 0.15 | (18.93, 25.89) | 27.72 | 0.72 | (2.91, 14.96) | 1011.69 | -229.17 | (11.58,13.49) | 14.01 | -0.13 |
|  |  | (7.91,12,84) |  |  | 52.86~14.23 |  |  | 21.86~6.96 |  |  | 8.62~12.05 |  |  | 12.56~1.91 |  |  |
| LB453 | 919.5 | 1.4~17.4 | 1521.6 | -379.92 | (42.33, 60.78) | 17.29 | -0.29 | (17.47, 31.56) | 47.77 | -1.51 | (3.16, 15.94) | 37.22 | 4.51 | (11.04,13.89) | 9.81 | -0.19 |
|  |  | (8.2,16) |  |  | 50.71~18.45 |  |  | 24.10~14.09 |  |  | 8.03~12.78 |  |  | 12.55~2.85 |  |  |
| LB411 | 846.2 | 0.8~8.5 | 1146.5 | 466.68 | (50.94, 56.92) | 3.30 | -0.03 | (16.25, 24.95) | 20.72 | 0.68 | (5.49, 9.89) | 2.45 | -0.58 | (11.62,13.42) | 8.21 | -0.01 |
|  |  | (3.61,7.7) |  |  | 54.43~5.98 |  |  | 21.14~8.70 |  |  | 7.70~4.40 |  |  | 12.47~1.80 |  |  |
| LB412 | 854.6 | 0.5~11.2 | 1477.9 | 26.15 | (44.21, 59.88) | 21.40 | 0.59 | (11.65, 30.20) | 57.84 | -9.37 | (3.37, 16.80) | 357.89 | 27.91 | (10.40,13.60) | 9.23 | 0.09 |
|  |  | (4.07,7.24) |  |  | 51.58~15.67 |  |  | 23.58~18.55 |  |  | 8.02~13.43 |  |  | 12.22~3.20 |  |  |
| LB421 | 830.2 | 0.5~17.9 | 2730.7 | -319.15 | (45.01, 62.53) | 14.15 | -0.01 | (14.61, 31.39) | 112.19 | 1.44 | (4.91, 9.45) | 95.87 | 1.73 | (11.71,14.76) | 5.50 | 0.01 |
|  |  | (5.48,15,51) |  |  | 53.75~17.52 |  |  | 21.58~16.78 |  |  | 6.71~4.54 |  |  | 13.24~3.05 |  |  |
| LZ320 | 860.6 | 1.55~20.5 | 1819.1 | 511.07 | (51.72, 63.99) | 12.92 | -0.39 | (14.95, 22.4) | 55.01 | -4.18 | (3.79, 11.27) | 653.23 | 90.77 | (11.20,14.38) | 38.34 | 0.52 |
|  |  | (7.44,18.95) |  |  | 57.80~12.27 |  |  | 18.55~7.45 |  |  | 6.47~7.48 |  |  | 12.82~3.18 |  |  |
| LZ336 | 845.0 | 1~20.4 | 186.21 | 72.54 | (54.44, 63.92) | 4.15 | -0.02 | (13.35, 20.52) | 101.30 | -0.90 | (4.05, 8.19) | 264.98 | 1.09 | (11.30,14.00) | 23.74 | -0.63 |
|  |  | (6.94,19.4) |  |  | 59.44~9.48 |  |  | 17.37~7.17 |  |  | 6.13~4.14 |  |  | 13.14~2.70 |  |  |
| XXI-3 | 782.8 | 0.8~19.1 | 1212.8 | 233.76 | (50.09, 62.97) | 14.07 | -0.22 | (12.78, 21.68) | 148.40 | -6.84 | (4.18, 8.9) | 171.00 | 16.85 | (12.44,14.34) | 8.84 | -0.03 |
|  |  | (7.93, 18.3) |  |  | 58.15~12.88 |  |  | 17.94~8.90 |  |  | 5.42~4.72 |  |  | 13.27~1.90 |  |  |
| XXI-9 | 789.1 | 0.5~17.35 | 2389.5 | 96.22 | (48.25, 60.95) | 0.16 | 0.10 | (13.86, 25.59) | 63.76 | -5.52 | (2.90, 11.11) | 224.23 | -48.29 | (12.18,13.83) | 5.24 | 0.02 |
|  |  | (5.02, 16.85) |  |  | 55.17~12.70 |  |  | 20.69~11.73 |  |  | 6.34~9.21 |  |  | 13.02~1.90 |  |  |
| XXII-13 | 793.0 | 0.5~15.85 | 1749.7 | 58.67 | (51.84, 66.17) | 5.48 | -0.12 | (12.29, 23.44) | 56.55 | -1.43 | (2.30, 11.39) | 286.97 | 18.95 | (12.20,14.60) | 3.55 | -0.03 |
|  |  | (4.62, 15.35) |  |  | 59.63~14.33 |  |  | 18.09~11.15 |  |  | 4.75~9.09 |  |  | 13.49~2.40 |  |  |
| XXII-15 | 816.7 | 0.7~16.6 | 2250. 9 | -51.75 | (49.17, 63.68) | 8.76 | -0.10 | (12.95, 23.50) | 37.83 | -3.47 | (2.61, 15.60) | 154.31 | 47.08 | (11.02,14.32) | 23.28 | 3.48 |
|  |  | (5.33, 15.9) |  |  | 58.18~14.51 |  |  | 19.59~10.55 |  |  | 5.86~12.99 |  |  | 13.10~3.30 |  |  |
| XXII-18 | 806.9 | 0.9~12.35 | 2697.5 | 213.71 | (52.43, 63.03) | 10.05 | 0.12 | (12.12, 21.20) | 86.59 | -7.18 | (5.00, 11.03) | 149.78 | -0.43 | (12.30,13.98) | 7.27 | -0.04 |
|  |  | (4.22, 11.45) |  |  | 57.71~10.60 |  |  | 17.34~9.08 |  |  | 7.67~6.03 |  |  | 13.09~2.40 |  |  |
| XX20 | 808.4 | 0.7~19.88 | 4354.3 | 93.60 | (50.07, 67.58) | 8.83 | 0.15 | (9.73, 25.50) | 175.83 | -18.3 | (2.10, 10.75) | 28.53 | 1.02 | (11.61,14.23) | 9.70 | 0.01 |
|  |  | (3.86, 19.18) |  |  | 58.42~17.51 |  |  | 17.58~15.77 |  |  | 7.09~8.65 |  |  | 13.03~2.62 |  |  |
| XXIII-4 | 809.2 | 1.6~10.3 | 970.19 | -179.07 | (54.69, 65.04) | 8.39 | -0.12 | (14.08, 21.01) | 29.65 | -2.60 | (2.79, 7.87) | 305.98 | 6.47 | (12.60,14.93) | 3.43 | -0.07 |
|  |  | (6.29, 8.7) |  |  | 60.02~10.35 |  |  | 17.91~6.93 |  |  | 5.19~5.08 |  |  | 13.76~2.33 |  |  |
| XXIII-8 | 835.4 | 0.5~11.08 | 1147.1 | 262.20 | (52.95, 66.95) | 0.05 | -0.01 | (9.00, 26.06) | 23.72 | 2.84 | (4.92, 11.16) | 140.61 | 31.63 | (12.61,14.33) | 4.78 | -0.06 |
|  |  | (3.14, 10.58) |  |  | 60.59~14.00 |  |  | 16.17~17.06 |  |  | 6.60~6.24 |  |  | 13.60~1.72 |  |  |
| LDD16 | 873.3 | 1.9~16 | 523.79 | -72.53 | (54.85, 62.23) | 8.51 | -0.14 | (16.87, 24.49) | 51.23 | 1.84 | (1.75, 4.89) | 569.08 | 89.17 | (11.84,13.91) | 1.91 | -0.06 |
|  |  | (9.81, 14.1) |  |  | 59.15~7.38 |  |  | 19.71~7.62 |  |  | 3.12~3.14 |  |  | 13.03~2.07 |  |  |
| LDD18 | 836.8 | 1~21.7 | 1283.3 | -123.98 | (55.78, 64.73) | 4.05 | 0.00 | (13.01, 23.95) | 40.04 | -0.72 | (2.59, 7.46) | 214.15 | 9.32 | (11.56,14.09) | 8.28 | -0.26 |
|  |  | (9.04, 20.7) |  |  | 59.89~7.38 |  |  | 18.47~10.94 |  |  | 4.28~4.87 |  |  | 13.28~2.53 |  |  |
| LDD43 | 820.3 | 3~21 | 149.90 | 9.67 | (50.14, 60.95) | 18.94 | -0.13 | (11.72, 21.23) | 63.26 | -3.80 | (4.62, 14.39) | 316.90 | 9.40 | (11.66,13.76) | 8.32 | -0.18 |
|  |  | (7.72, 18) |  |  | 56.32~10.81 |  |  | 16.65~9.51 |  |  | 8.93~9.77 |  |  | 13.07~2.10 |  |  |
| XXII-10 | 888.1 | 1.1~13.05 | 1194.6 | 216.57 | (54.47, 62.10) | 3.24 | -0.03 | (14.44, 23.52) | 79.45 | 1.79 | (2.32, 9.08) | 561.04 | -63.13 | (11.69,13.57) | 9.74 | -0.08 |
|  |  | (5.62, 11.95) |  |  | 58.43~7.63 |  |  | 19.28~9.08 |  |  | 5.48~6.76 |  |  | 12.64~1.88 |  |  |
| XXII-9 | 927.9 | 0.5~16.75 | 5049.2 | -643.75 | (54.49, 63.55) | 1.05 | -0.03 | (14.55, 27.14) | 6.52 | 0.64 | (1.42, 3.40) | 187.25 | -9.49 | (11.94,13.77) | 2.74 | -0.02 |
|  |  | (4.85, 16.25) |  |  | 59.00~9.06 |  |  | 21.94~12.59 |  |  | 2.16~1.98 |  |  | 12.70~1.83 |  |  |
| XXII-4 | 858.1 | 0.95~13.1 | 694.69 | -35.86 | (52.14, 59.35) | 1.92 | -0.01 | (19.47, 26.01) | 24.35 | -0.11 | (1.62, 6.23) | 1648.26 | -113.67 | (11.90,13.68) | 0.09 | -0.01 |
|  |  | (5.91, 12.15) |  |  | 55.86~7.21 |  |  | 23.11~6.54 |  |  | 3.60~4.61 |  |  | 12.63~1.78 |  |  |
| XXIII-13 | 826.0 | 0.7~14.2 | 624.45 | 79.07 | (55.06, 64.58) | 5.15 | -0.10 | (13.88, 20.87) | 12.48 | -0.36 | (1.44, 6.62) | 444.75 | 40.45 | (12.79,14.23) | 2.18 | -0.02 |
|  |  | (5.98, 13.5) |  |  | 60.56~9.52 |  |  | 17.93~6.99 |  |  | 3.88~5.18 |  |  | 13.57~1.78 |  |  |
| XXIII-15 | 843.9 | 0.65~14.75 | 1640.8 | -195.61 | (55.64, 64.70) | 7.45 | 0.02 | (12.50, 21.30) | 76.99 | -7.01 | (2.02, 6.79) | 252.58 | -2.33 | (12.10,14.80) | 7.49 | 0.10 |
|  |  | (5.96, 14.1) |  |  | 61.00~9.06 |  |  | 17.33~9.80 |  |  | 3.94~4.77 |  |  | 13.22~2.70 |  |  |
| QY714 | 842.4 | 2.3~15.63 | 992.02 | -157.36 | (47.81, 56.71) | 0.40 | -0.03 | (15.03, 31.23) | 33.69 | 3.80 | (4.17, 11.23) | 59.51 | -8.17 | (10.07,14.05) | 46.58 | -0.16 |
|  |  | (8.47,13.33) |  |  | 53.04~8.90 |  |  | 23.33~16.20 |  |  | 6.89~7.06 |  |  | 12.13~3.98 |  |  |
| QY718 | 883.6 | 2~21.07 | 1287.8 | -328.59 | (44.12, 58.59) | 2.36 | 0.33 | (18.55, 29.71) | 24.55 | 2.01 | (7.07, 16.79) | 93.52 | 3.97 | (9.7,12.92) | 13.25 | -0.18 |
|  |  | (10.88,19.07) |  |  | 49.99~14.47 |  |  | 23.13~11.16 |  |  | 10.90~7.72 |  |  | 11.54~3.22 |  |  |
| QY719 | 988.3 | 0.9~20.22 | 1356.7 | -268.75 | (48.12, 62.88) | 2.10 | -0.02 | (19.05, 26.91) | 44.42 | -3.03 | (1.88, 10.42) | 370.77 | 25.31 | (10.18,14.47) | 2.23 | 0.07 |
|  |  | (7.74,19.32) |  |  | 55.64~14.47 |  |  | 22.75~7.86 |  |  | 4.43~9.54 |  |  | 12.70~3.22 |  |  |
| QY721 | 934.0 | 2.65~13.64 | 798.68 | 133.05 | (52.16, 57.79) | 3.66 | -0.03 | (21.02, 27.72) | 9.18 | 0.00 | (1.40, 7.12) | 715.24 | 111.89 | (10.84,13.65) | 18.33 | -0.32 |
|  |  | (6.56,19.07) |  |  | 55.54~5.63 |  |  | 24.39~6.70 |  |  | 2.88~5.72 |  |  | 12.61~2.81 |  |  |
| QY734 | 898.3 | 2.2~16.45 | 991.42 | -115.96 | (54.45, 61.27) | 4.14 | 0.03 | (18.01, 23.52) | 75.57 | -4.96 | (2.42, 6.18) | 51.99 | -4.88 | (11.58,14.41) | 23.59 | 0.02 |
|  |  | (8.44,14.25) |  |  | 57.33~6.82 |  |  | 20.62~5.51 |  |  | 4.81~3.76 |  |  | 12.95~2.83 |  |  |
| QY739 | 905.2 | 1.93~22.4 | 1504.3 | -374.15 | (40.84, 55.98) | 30.55 | 0.16 | (18.37, 32.00) | 81.84 | -3.39 | (5.13, 16.47) | 388.19 | -21.71 | (9.35,13.18) | 31.87 | -0.58 |
|  |  | (9.77, 20.57) |  |  | 47.14~15.14 |  |  | 25.66~15.63 |  |  | 11.32~11.34 |  |  | 11.13~3.85 |  |  |
| QY740 | 890.6 | 1.5~21.83 | 1404.2 | -528.91 | (41.5, 57.74) | 1.21 | 0.68 | (17.30, 29.67) | 75.84 | -6.65 | (7.35, 14.03) | 32.15 | 6.50 | (9.99,13.02) | 20.46 | 0.68 |
|  |  | (10.08,20.33) |  |  | 50.63~16.24 |  |  | 23.45~12.37 |  |  | 9.99~6.68 |  |  | 11.31~3.03 |  |  |
| XXVI-10 | 827.8 | 2.8~23.69 | 919.61 | -114.71 | (53.11, 62.69) | 13.01 | 0.23 | (11.86, 24.45) | 143.49 | -22.39 | (6.04, 9.40) | 114.21 | -2.05 | (11.27,13.16) | 6.87 | 0.04 |
|  |  | (11.46, 0.89) |  |  | 56.10~13.66 |  |  | 20.05~13.59 |  |  | 7.58~3.36 |  |  | 12.09~1.89 |  |  |
| XX21 | 878.2 | 0.8~21.98 | 2684.7 | -1147.8 | (48.20, 57.43) | 7.31 | 0.02 | (16.35, 28.67) | 40.33 | -1.97 | (2.21, 8.36) | 51.22 | -21.31 | (10.37,13.38) | 22.29 | 0.28 |
|  |  | (11.59,21.18) |  |  | 52.58~9.23 |  |  | 24.29~12.32 |  |  | 6.67~3.15 |  |  | 11.57~3.01 |  |  |
| XXVI-25 | 921.5 | 0.8~22.4 | 2234.7 | -166.31 | (50.86, 57.62) | 2.71 | -0.02 | (16.63, 28.22) | 51.97 | -0.97 | (1.60, 7.21) | 322.09 | -58.54 | (11.88,13.49) | 3.84 | -0.04 |
|  |  | (7.39, 21.6) |  |  | 54.63~6.76 |  |  | 23.23~11.59 |  |  | 4.14~5.61 |  |  | 12.83~1.61 |  |  |
| XXVI-26 | 878.6 | 2.35~19.66 | 996.66 | -159.30 | (47.70, 58.05) | 3.52 | -0.06 | (17.10, 27.65) | 37.30 | -1.12 | (3.36, 7.33) | 287.35 | -5.29 | (11.70,13.48) | 5.05 | -0.06 |
|  |  | (10.82,17.31) |  |  | 53.14~10.35 |  |  | 23.34~10.55 |  |  | 5.38~3.97 |  |  | 12.68~1.78 |  |  |
| XXVI-27 | 955.8 | 1.5~17.59 | 1392.7 | -134.36 | (52.14, 60.00) | 3.74 | 0.05 | (16.15, 27.15) | 94.45 | -4.99 | (2.22, 6.79) | 315.44 | -28.14 | (11.93,14.09) | 12.33 | 0.19 |
|  |  | (6.94, 16.09) |  |  | 55.46~7.86 |  |  | 22.65~11.00 |  |  | 4.06~3.97 |  |  | 12.70~2.06 |  |  |
| XXVI-33 | 861.6 | 0.9~20 | 2148.6 | -833.55 | (47.94, 59.65) | 8.11 | -0.09 | (18.65, 29.35) | 64.05 | 0.61 | (1.82, 10.06) | 283.19 | -58.77 | (10.57,12.64) | 7.17 | -0.06 |
|  |  | (9.39, 19.1) |  |  | 55.02~7.86 |  |  | 23.58~10.70 |  |  | 4.72~8.24 |  |  | 11.65~2.07 |  |  |
| XXVI-34 | 904.7 | 1.3~16.83 | 2100.1 | -742.12 | (53.35, 57.76) | 0.07 | -0.01 | (21.28, 28.60) | 28.76 | -0.01 | (1.70, 6.10) | 901.51 | 16.78 | (10.82,12.82) | 3.12 | -0.01 |
|  |  | (9.00, 15.51) |  |  | 55.70~4.41 |  |  | 24.06~17.32 |  |  | 3.65~4.40 |  |  | 11.77~2.00 |  |  |
| XXVI-48 | 1023.7 | 1.09~14.4 | 1018.3 | -276.55 | (52.26, 57.97) | 3.58 | 0.04 | (21.25, 28.50) | 26.19 | -0.10 | (2.01, 6.18) | 484.17 | 100.25 | (11.77,13.75) | 0.33 | 0.02 |
|  |  | (7.06, 13.31) |  |  | 54.79~5.71 |  |  | 24.44~7.25 |  |  | 3.28~4.17 |  |  | 12.43~1.98 |  |  |
| XXVII-1 | 919.0 | 0.8~14.28 | 538.97 | 42.94 | (47.47, 59.62) | 12.63 | -0.35 | (16.91, 26.99) | 1.46 | 0.03 | (2.36, 15.59) | 663.95 | -14.01 | (9.73,13.16) | 26.40 | -0.36 |
|  |  | (6.23, 13.48) |  |  | 54.14~12.15 |  |  | 22.49~10.08 |  |  | 7.38~13.23 |  |  | 11.46~3.43 |  |  |
| XXV-13 | 806.4 | 0.7~14.6 | 3742.6 | -1896.7 | (51.54, 59.19) | 5.00 | -0.07 | (18.41, 25.74) | 30.24 | 0.83 | (2.12, 8.06) | 185.96 | 16.64 | (12.30,13.89) | 7.55 | -0.03 |
|  |  | (9.26, 13.9) |  |  | 55.98~7.65 |  |  | 21.42~7.33 |  |  | 4.66~6.94 |  |  | 13.02~1.59 |  |  |
| XXV-28 | 814.5 | 2~12.3 | 1026.3 | -263.58 | (54.36, 59.69) | 3.08 | 0.00 | (18.84, 26.16) | 38.28 | -0.09 | (1.96, 7.63) | 654.06 | -50.11 | (12.63,14.09) | 2.08 | 0.00 |
|  |  | (7.98, 10.3) |  |  | 56.60~5.33 |  |  | 21.70~7.32 |  |  | 4.57~5.67 |  |  | 13.43~1.46 |  |  |
| XXV-36 | 835.6 | 3.4~16.3 | 941.76 | -46.76 | (47.41, 54.95) | 2.89 | 0.00 | (20.69, 26.05) | 15.10 | 0.09 | (6.40, 10.87) | 40.21 | -2.70 | (11.64,13.27) | 1.93 | 0.04 |
|  |  | (9.15, 12.9) |  |  | 51.33~7.54 |  |  | 23.28~5.36 |  |  | 9.01~4.47 |  |  | 12.26~1.63 |  |  |
| XXVI-4 | 793.4 | 0.65~14.03 | 1782.5 | 448.52 | (42.44, 58.95) | 28.72 | -1.18 | (11.48, 31.70) | 156.33 | -12.08 | (4.20, 15.24) | 148.24 | 7.62 | (10.94,14.86) | 13.07 | -0.15 |
|  |  | (5.20, 12.9) |  |  | 53.12~16.51 |  |  | 21.25~20.22 |  |  | 7.78~11.04 |  |  | 13.06~1.63 |  |  |
| XXVI-7 | 831.3 | 0.69~16.99 | 1258.6 | -447.95 | (45.23, 58.89) | 11.14 | -0.37 | (17.37, 29.78) | 51.75 | 0.50 | (2.01, 8.33) | 443.69 | -83.76 | (11.62,14.17) | 4.79 | 0.01 |
|  |  | (8.71, 16.39) |  |  | 54.88~13.66 |  |  | 23.20~12.41 |  |  | 5.27~6.32 |  |  | 13.04~2.55 |  |  |
| DD315 | 810.0 | 1.5~19.05 | 643.69 | 78.94 | (42.42, 60.24) | 23.85 | 0.34 | (14.74, 33.17) | 111.96 | -8.01 | (7.08, 16.13) | 86.91 | -4.19 | (9.68,13.68) | 32.17 | 0.34 |
|  |  | (9.73, 17.55) |  |  | 48.08~17.82 |  |  | 25.33~18.43 |  |  | 10.20~9.05 |  |  | 11.31~4.00 |  |  |
| DD330 | 796.6 | 1.0~15.7 | 1944.6 | -158.51 | (41.21, 61.92) | 39.26 | 2.15 | (9.65, 35.92) | 324.26 | 7.06 | (6.41, 15.20) | 248.59 | -33.12 | (11.25,13.43) | 36.84 | -0.51 |
|  |  | (8.86, 14.7) |  |  | 46.65~20.71 |  |  | 24.79~18.43 |  |  | 11.32~8.79 |  |  | 12.04~2.18 |  |  |
| DD335 | 802.3 | 1.1~14.4 | 724.23 | -287.10 | (41.02, 55.42) | 10.40 | -0.20 | (20.01, 30.08) | 29.07 | -1.65 | (4.44, 14.41) | 111.89 | 4.92 | (9.30,13.30) | 11.35 | -0.03 |
|  |  | (8.42, 13.3) |  |  | 50.60~14.40 |  |  | 25.48~10.07 |  |  | 7.75~9.97 |  |  | 11.06~4.00 |  |  |

Table S2 Results of principal component analysis of elevation and orebody parameters for 9,007 wells, high- and low-elevation wells, thick single orebody and thin single orebody XX20 in the western Guangxi, China

|  | PCA for 9007 exploration wells | | | PCA for high-elevation wells | | | PCA for low-elevation wells | | | PCA for thick orebody XX21 | | | PCA for thin orebody XX20 | | |
| --- | --- | --- | --- | --- | --- | --- | --- | --- | --- | --- | --- | --- | --- | --- | --- |
|  | 1 | 2 | 3 | 1 | 2 | 3 | 1 | 2 | 3 | 1 | 2 | 3 | 1 | 2 | 3 |
| Elevation | -0.06 | 0.04 | **0.95** | 0.05 | -0.19 | **0.72** | -0.46 | 0.39 | -0.17 | -0.06 | -0.20 | **-0.81** | -0.17 | -0.02 | **0.65** |
| Thickness | **-0.63** | 0.18 | -0.21 | -0.42 | 0.00 | -0.12 | -0.11 | -0.07 | **0.82** | -0.15 | 0.06 | **0.78** | -0.10 | -0.23 | **0.85** |
| Ore-bearing rate | -0.28 | 0.48 | 0.20 | -0.03 | 0.30 | **0.70** | -0.03 | 0.21 | **0.69** | -0.25 | -0.13 | 0.52 | 0.10 | 0.45 | 0.54 |
| Al_2_O_3_ | **0.81** | 0.52 | -0.07 | **0.75** | **0.62** | 0.01 | **0.83** | 0.49 | 0.01 | **0.89** | 0.25 | -0.27 | **0.83** | 0.44 | -0.04 |
| SiO_2_ | -0.24 | **-0.88** | 0.04 | -0.16 | **-0.94** | 0.00 | -0.21 | **-0.90** | -0.13 | 0.07 | **-0.96** | -0.19 | 0.10 | **-0.96** | 0.04 |
| Fe_2_O_3_^T^ | **-0.93** | -0.04 | 0.08 | **-0.96** | 0.00 | 0.04 | **-0.93** | -0.07 | 0.12 | **-0.97** | 0.21 | 0.08 | **-0.98** | 0.02 | 0.06 |
| LOI | **0.67** | 0.19 | -0.26 | 0.58 | 0.24 | -0.36 | **0.70** | 0.19 | -0.11 | **0.65** | 0.03 | 0.57 | **0.81** | -0.23 | -0.19 |
| A/S | 0.19 | **0.88** | -0.07 | 0.09 | **0.92** | 0.01 | 0.24 | **0.86** | 0.11 | 0.02 | **0.96** | -0.06 | 0.20 | **0.90** | -0.11 |
| A/F | **0.91** | 0.15 | -0.11 | **0.94** | 0.21 | -0.04 | **0.92** | 0.14 | -0.09 | **0.97** | -0.08 | -0.14 | **0.95** | 0.18 | -0.08 |
| E | 3.86 | 1.71 | 1.01 | 3.70 | 1.60 | 1.12 | 3.76 | 1.70 | 1.05 | 3.27 | 2.21 | 1.73 | 3.60 | 2.09 | 1.38 |
| % | 42.89 | 19.02 | 11.17 | 41.07 | 17.74 | 12.47 | 41.80 | 18.85 | 11.72 | 36.31 | 24.53 | 19.21 | 39.98 | 23.27 | 15.38 |
| Cum.% | 42.89 | 61.91 | 73.08 | 41.07 | 58.81 | 71.28 | 41.80 | 60.66 | 72.37 | 36.31 | 60.84 | 80.05 | 39.98 | 63.25 | 78.62 |

Rotation method: Varimax with Kaiser Normalization. E,% and Cum. % denote the Eigenvalues, % of variance and cumulative% of variance, respectively. Bold values represent values above threshold (0.60 in this study)
